# Prognostic factors of second hematopoietic allogeneic stem cell transplantation among hematological malignancy patients relapsed after first hematopoietic stem cell transplantation: A single center study

**DOI:** 10.3389/fimmu.2022.1066748

**Published:** 2023-01-04

**Authors:** Yue Lu, Jian-Ping Zhang, Yan-Li Zhao, Min Xiong, Rui-Juan Sun, Xing-Yu Cao, Zhi-Jie Wei, Jia-Rui Zhou, De-Yan Liu, Jun-Fang Yang, Xian Zhang, Dao-Pei Lu, Peihua Lu

**Affiliations:** ^1^ Department of Bone Marrow Transplantation, Hebei Yanda Lu Daopei Hospital, Langfang, China; ^2^ Department of Hematology and Immunology, Hebei Yanda Lu Daopei Hospital, Langfang, China; ^3^ Beijing Lu Daopei Institute of Hematology, Beijing, China

**Keywords:** second allogeneic hematopoietic stem cell transplant, relapse, prognosis factor, hematological malignancy, first allogeneic hematopoietic stem cell transplantation

## Abstract

**Introduction:**

We aimed to evaluate prognostic factors of a second allogeneic stem cell transplantation (allo-HSCT2) among hematological malignancy patients who have relapsed after the first allo-HSCT(allo-HSCT1).

**Methods:**

We retrospectively analyzed 199 hematological malignancy patients who received allo-HSCT2 as a salvage treatment post allo-HSCT1 relapse between November 2012 and October 2021.

**Results:**

The median age at allo-HSCT2 was 23 (range: 3-60) years. The median time to relapse after HSCT1 was 9 (range: 1-72) months. Prior to allo-HSCT2, patients had the following hematopoietic cell transplantation-comorbidity indexes (HCT-CI): 127 with a score of 0, 52 with a score of 1, and 20 with a score of 2 or greater. Fifty percent of patients received chimeric antigen receptor (CAR) T-cell therapy following HSCT1 relapse. Disease status was minimal residual disease (MRD)-negative complete remission (CR) among 119 patients, MRD-positive CR among 37 patients and non-remission (NR) for 43 patients prior to allo-HSCT2. Allo-HSCT2 was performed from a new donor in 194 patients (97.4%) and 134 patients (67.3%) received a graft with a new mismatched haplotype. The median follow-up time was 24 months (range: 6-98 months), and the 2-year OS and LFS were 43.8% ± 4.0% and 42.1% ± 4.1%, respectively. The 2-year cumulative incidence of relapse (CIR) and non-relapse mortality (NRM) was 30.0%±4.8% and 38.5%±3.8%, respectively. Cox regression multivariate analysis showed that disease statusof MRD-negative CR, HCT-CI score of 0 prior to allo-HSCT2, and new mismatched haplotype donor were predictive factors of improved OS and LFS compared to patients without these characteristics. Based on these three favorable factors, we developed a predictive scoring system for patients who received allo-HSCT2. Patients with a prognostic score of 3 who had the three factors showed a superior 2-year OS of 63.3% ± 6.7% and LFS of 63.3% ± 6.7% and a lower CIR of 5.5% ± 3.1% than patients with a prognostic score of 0. Allo-HSCT2 is feasible and patients with good prognostic features prior to allo-HSCT2 —disease status of CR/MRD- and HCT-CI score of 0 as well as a second donor with a new mismatched haplotype could have the maximal benefit from the second allo-HSCT.

**Conclusions:**

Allo-HSCT2 is feasible and patients with good prognostic features prior to allo-HSCT2 —disease status of CR/MRD- and HCT-CI score of 0 as well as a second donor with a new mismatched haplotype could have the maximal benefit from the second allo-HSCT.

## Introduction

Relapse of a primary hematological malignancy is the most common cause of hematopoietic stem cell transplant (HSCT) failure and is associated with poor prognosis (1–3). Currently, there are no established treatment guidelines for these patients and strategies for withdrawal of immunosuppression, donor lymphocyte infusion (DLI), and re-induction chemotherapy vary among institutions (4) (5). A second allogeneic transplant is also a frequently employed strategy for these patients, and it is assumed that by switching the transplant donor immune system, and different graft versus tumor effects (GVT) may develop. However, a second HSCT might be limited due to a high risk of both relapse and non-relapse mortality (NRM). There are few data to support a second allogeneic HSCT (allo-HSCT2) over DLI (6–8). Reports from the EBMT and CIBMTR have described pediatric and adults who have relapsed following a first allo-HSCT (allo-HSCT1) and underwent an allo-HSCT2. These studies reported 5-year leukemia-free survival (LFS) ranging between 7% and 25% following an allo-HSCT2 (9–12). Several prior studies have identified factors associated with overall survival among allo-HSCT2 recipients including1) age, 2) interval time from allo-HSCT1 to relapse, 3) allo-HSCT1 conditioning regimen, 4) Karnofsky score at allo-HSCT2, 5) allo-HSCT2 donor type, 6) occurrence of acute and chronic graft-versus-host disease (aGVHD and cGVHD) following the allo-HSCT1.

Despite these studies, questions on optimal allo-HSCT2 donor selection remain including whether changing to a different donor or using the original donor is beneficial Thus far, there is emerging evidence that an HSCT from readily available alternative donor sources, such as HLA-haploidentical family donors, can lead to long-term survival (13–16). One study showed that an allograft with a new mismatched haplotype may improve outcomes following a second bone marrow transplant among relapsed hematologic malignancies, yet the study was limited by a small size and lack of similar results from other research centers (17).

To better understand whether or not and how to perform a second allo-HSCT successfully on relapsed hematological malignancy patients who have undergone a first allo-HSCT and to further understand the prognosis status of these difficult-to-treat patients, we respectively analyzed 199 hematological malignancy patients receiving an allo-HSCT2 following allo-HSCT1 relapse.

## Materials and methods

### Patient eligibility criteria and donor selection

This was a retrospective, single-center study conducted at the Department of Bone Marrow Transplantation at the Hebei Yanda Lu Daopei Hospital in Langfang, China. Inclusion criteria consisted of patients diagnosed with a malignant hematological disorder, a history of disease relapse after an HSCT1 and a subsequent allo-HSCT2 between November 2012 and October 2021. HLA data (a high resolution of HLA- A, B, Cw, DRB1, DQ-loci) was obtained for all HSCT recipients and their donors. For analysis purposes, donors were categorized as matched or haploidentical. A matched sibling donor (MSD) or matched unrelated donor (MUD) or umbilical cord blood donor (UCBD) with one or two HLA mismatched were categorized as “matched”.

Patients were evaluated using the Hematopoietic cell transplantation-comorbidity indexes (HCT-CI) prior to allo-HSCT2 (18). All patients were treated according to clinical protocols approved by the Institutional Review Board of Hebei Yanda Lu Daopei Hospital. The study protocol was approved by the Ethics Committee at Hebei Yanda Lu Daopei Hospital. Informed consent was obtained from all patients, and the study was conducted according to the Declaration of Helsinki.

### Conditioning regimen and evaluation of related organ toxicity

All patients received total body irradiation (TBI)-based, Busulfan (Bu)-based or melphalan (Mel)-based myeloablative conditioning regimen (MAC). Our standardized MAC consisted of the following: high dose-cytarabine (HD-Ara-c) (IV, 2–3 g/m^2^ per day) for 3 days (days − 12~ − 10), Bu (IV, 3.2 mg/kg per day) for 4 days (days − 9~− 6) or TBI (shielding eyes and lungs, 400cGy per day) for 3 days (days − 9~− 7) or Mel (IV 70mg/m^2^ per day) for 2 days (days − 3~− 2), cyclophosphamide(Cy) (1.8 g/m^2^ per day) for 2 days (days − 5~− 4) or fludarabine(Flu) (30mg/m^2^/per day) for 5 days (days − 6~− 2), Semustine (Me-CCNU) (250 mg/m^2^) for 1 day (day − 3), and Antihuman T-lymphocyte globulin(ATG) for 4 days (days − 5~ − 2) [either as ATG thymoglobuline (ATG-T) at a total dose of 5.0~8 mg/kg(n=72), or ATG-Fresenius (ATG-F) at a total dose of 15-20 mg/kg (n=101) or ATG- Porcine Immunoglobulin (ATG-P) 80-100mg/kg(n=20)]. As previously described, part of the scheme has been modified (19–21).

Conditioning related to organ toxicity was evaluated according to NCI-CTCAE.5.0.

### Study end points, definitions, and assessments

Relapse and death from any cause were considered events. The primary end point of the study was LFS. Secondary end points included treatment- related mortality (TRM), cumulative incidence of relapse (CIR), overall survival (OS), neutrophil engraftment, and acute and chronic graft vs host disease (GVHD). LFS was calculated from the date of second transplant until relapse, death, or the last disease-free follow-up. OS was calculated from the date of second transplant until death or the last follow-up. TRM was defined as death without prior relapse. Hematologic relapse was defined by the reappearance of blasts in the peripheral blood (PB), any manifestation of leukemia outside the hematopoietic system, or bone marrow (BM) infiltration higher than 5% of blasts in a representative smear. Acute GVHD and chronic GVHD were graded according to standard criteria (22, 23). On the 28th day, disease response and chimerism were assessed in PB and BM. Chimerism detection was carried out by short tandem repeat analysis among peripheral CD3+ cells and in unfractionated BM. Ninety-five percent of donor cell defined full donor chimerism.

### Stem cell source and graft-versus-host disease prophylaxis

Unmanipulated stem cells were used for all patients. All patients received granulocyte colony–stimulating factor (G-CSF)-primed allogeneic bone marrow combined with peripheral blood stem cell (PBSC) or PBSC. The target number of mononuclear cells in total was ≥ 5 × 10^8^/kg and CD34+ cells ≥ 2 × 10^6^/kg. GVHD prophylaxis included mycophenolate mofetil (MMF), and cyclosporine-A(CSA) with short-term methotrexate. CSA was tapered gradually (1/5 every 2 weeks) from day 45 until the CSA was ceased, if no GVHD was detected. CSA was stopped whenever hematology recurrence or minimal residual disease (MRD) was detected.

### Statistical analysis

The probability of OS and LFS were calculated using Kaplan–Meier estimates. Cumulative incidence of TRM and CIR were calculated to accommodate for competing risks and results were presented according to the Fine and Gray model. Log-rank and Breslow tests were used for univariate comparisons for all variables considered. For the univariate analyses, we used the probability% (95% Confidence Index, 95%CI) for each subgroup. For multivariate analyses, we included all independent covariates with a p value <0.1 using the Cox proportional hazards regression model. For univariate analyses, the p-value was set at <0.05 for statistical significance. Statistical analyses were performed using the statistical package SPSS version 17 and R software version 3.4.1.

## Results

### Patients and transplant characteristics

A total of 199 hematological malignancy patients who had relapsed after HSCT1 and who received allo-HSCT2 between November 2012 and October 2021 were included in the analysis. Patient, disease, and HSCT characteristics are presented in **Table 1**.

**Table 1:** Patient, disease, and HSCT characteristics.

	Allo-HSCT1	Allo-HSCT2
Characteristics	199 (%)	199 (%)
Sex: male	144 (57.2%)	114 (57.2%)
Median age (range) at transplantation	23 (2-59)	23 (3-60)
Age <18 years	78 (39.1%)	79 (38.6%)
Diagnosis		
AML	72 (36.1%)	72 (37.4%)
ALL	108 (54.2%)	108 (54.2%)
B-ALL	96 (48.2%)	96 (48.2%)
T-ALL	12 (6.0%)	12 (6.0%)
AUL	7 (3.5%)	7 (3.5%)
MDS	6 (2.0%)	6 (2.0%)
CML	4 (2.0%)	4 (2.0%)
BPDCN	1 (0.5%)	1 (0.5%)
PIM	1 (0.5%)	1 (0.5%)
Median time from diagnosis to HSCT (mon.) (range)	20 (1-142)	29 (8-162)
Median time to relapse after HSCT1(mon.)(range)	NA	9 (2-72)
Median time between HSCT1 and HSCT2 (mon.) (range)	NA	16 (2-157)
Disease status at transplant		
CR 1/MFC-MRD positive	129 (64.8%)/26 (13.0%)	NA
≥CR2/MFC-MRD positive	40 (20.1%)/9 (4.5%)	156 (78.3%)/37 (18.5%)
Advanced status	30 (15.0%)	43 (21.1%)
Presence of TP53 mutation	13 (15.0%)	16 (7.8%)
Extramedullary disease before HSCT	22 (10.0%)	60 (30.1%)
Received therapy before allo-HSCT2		
Only stopped immunosuppressants	NA	3 (1.5%)
CT and/or targeted drug	NA	26 (13.0%)
CT and/or a targeted drug +DLI	NA	60 (30.1%)
CT and/or a targeted drug +CAR-T	NA	47 (23.6%)
CT and/or a targeted drug +DLI+CAR-T	NA	28 (14.0%)
Only CAR-T	NA	26 (13.0%)
Included radiotherapy	NA	9 (4.5%)
CAR-T following HSCT1 relapse	NA	101 (50.7%)
Donor type		
MSD	62 (31.1%)	4 (2.0%)
MUD	25 (12.5%)	34 (17.0%)
HID	105 (52.7%)	160 (80.4%)
UCBD	7 (3.5%)	1 (0.5%)
Occurrence of aGVHD post-HSCT1	NA	63 (31.1%)
I∼II aGVHD	NA	23 (11.5%)
III∼IV aGVHD	NA	39 (19.5%)
Occurrence of cGVHD post-HSCT1	NA	31 (15.2%)
Limited cGVHD	NA	13 (6.5%)
Extensive cGVHD	NA	18 (9.0%)
Donor gender matching		
M to M	51 (38.6%)	64 (32.1%)
M to F	26 (19.6%)	51 (25.6%)
F to F	23 (17.4%)	34 (17.0%)
F to M	22 (16.6%)	50 (25.1%)
HCT-CI pre-allo-HSCT2		
score 0	NA	127 (63.8%)
score 1	NA	52 (26.1%)
≥ score 2	NA	20 (10.0%)
Donor source in allo-HSCT2		
Parents	47 (23.6%)	103 (51.8%)
Sibling	82 (41.2%)	17 (8.5%)
Child	35 (17.6%)	32 (16.1%)
Collateral series	3 (1.5%)	12 (6.0%)
MUD	32 (16.1%)	35 (17.6%)
Allo-HSCT2 versus HSCT1 donor		
different		194 (97.4%)
with a new mismatched haplotype		134 (67.3%)
Conditioning regimen		
Myeloablative (with TBI-based)	37 (18.2%)	141 (69.4%)
Myeloablative (with BU-based)	149 (71.9%)	57 (28.0%)
Myeloablative (with Mel-based)	NA	5 (2.4%)
Unknown	17 (8.3%)	NA

ALL, Acute lymphoblastic leukemia; Allo-HSCT, Allogeneic hematopoietic stem cell transplantation; AML,Acute myeloid leukemia; AUL, Acute undifferentiated leukemia; MDS, Myelodysplastic syndrome; CML, Chronic myelogenous leukemia; BPDCN, Blastic plasmacytoid dendritic cell neoplasmsin; PIM, primary myelofibrosis (PIM);CI Confidence interval; CR,Complete remission; NR, non remission; MRD, Minimal residual disease; CAR-T, chimeric antigen receptor T-cell; MSD Matched sibling donor;MUD, matched unrelated donor; Auto, autologous; HID, haploidentical donor; UCBD, unrelated cord blood donor; CY, Cyclophosphamide; DLI, Donor lymphocyte infusions; HSCT Hematopoietic stem cell transplant; M, male; F, female; BU, Busulfan;TBI Total body irradiation; Mel, Melphalan; aGVHD, acute graft versus host disease; cGVHD, chronic graft versus host disease; HCT-CI, Hematopoietic Cell Transplantation Comorbidity Indexes; MFC, Multicolor flow cytometry; M, Men; F, Female; NA, not applicable.

Three patients ceased their immunosuppressants and had not received any other treatment, 26 patients had received chemotherapy and/or targeted drug therapy, 60 patients had received chemotherapy and/or a targeted drug plus DLI, 47 patients failed to respond to chemotherapy and/or a targeted drug and received chimeric antigen receptor (CAR) T-cell therapy, and 28 patients failed to respond to chemotherapy and/or a targeted drug plus DLI and received CAR-T cell therapy. Twenty-six patients received CAR-T cell therapy directly after allo-HSCT1 relapse, and nine patients received radiotherapy plus chemotherapy and/or targeted drug therapy.

One hundred and one patients received a total of 131 CAR-T cell infusions (**Table 2**). As described in previously published reports, we modified the CAR T-cell infusion. Almost all patients who received CAR-T cell therapy prior to allo-HSCT2 achieved CR (including 9 patients who were MRD+) except for 4 patients (2 AML patients failed to respond and received CD33+ and CD123+ CAR-T respectively; 2 B-ALL patients failed to respond and received CD19+CAR-T). The median time from completion of CAR-T therapy to an HID-HSCT2 was 60 days (range: 30-251 days).

**Table 2:** Information about CAR-T infusion following HSCT1 relapse.

CAR-T infusion	Type of CAR-T Cell	No. of Patients	Total CAR-T infusion
		101	131
Single CAR-T infusion		77	77
	CD19+	66	66
	CD7+	6	6
	CD123+	1	1
	CD33+	1	1
	CD19+CD22+	3	3
Second CAR-T infusion		21	42
	CD19+ →CD19+	10	20
	CD19+ → CD22+	6	12
	CD19+ → CD7+	1	2
	CD33+ →CD33+	1	2
	CD123+ →CD123+	1	2
	CD22+ →CD123+	1	2
	CD19+ →CD19+CD22+	1	2
Third CAR-T infusion	CD33+→CD33+→CD33+	1	3
Fourth CAR-T infusion	CD19+→CD19+→CD19+→CD19+	1	4
Fifth CAR-T infusion	CD19+→CD19+→CD19+→CD19+→CD19+	1	5

CAR-T, chimeric antigen receptor T-cell.

“→” means “changed to”.

Out of a total of 131 CAR-T cell infusions, the CAR-T cell sources were autologous cells in 91 cases (69.4%), allogeneic cells (first transplanted donor cells) in 23 cases (17.5%), and an unknown source in 17 cases (12.9%). A total of 156 patients achieved complete remission (CR) including 37 with MRD+CR and the remaining patients had non-remission (NR). The median interval time from allo-HSCT1 to allo-HSCT2 was 16 months (range: 24–157 months). The median interval time from relapse to allo-HSCT2 was 9 months(range:2–72) months.

### Patients and donor HLA-matching

**Figure 1:**
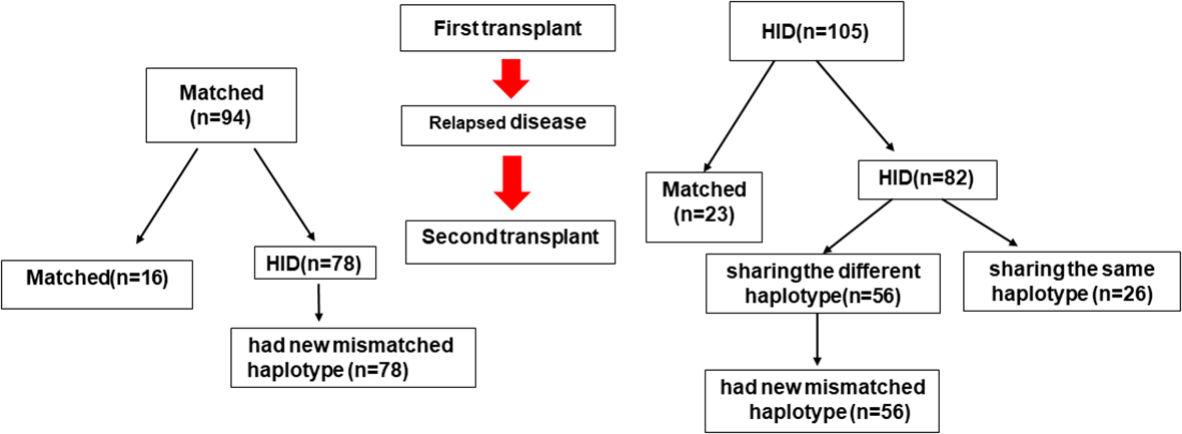
HLA-matching pathway. MSD, Matched sibling donor; MUD, matched unrelated donor; HID, haploidentical donor; UCB, unrelated cord blood donor; Auto, autologous.

Allo-HSCT2 was performed from different donors in 194 patients (97.4%). One hundred and five patients received their first allograft from a haploidentical donor, and of these, eighty-two received a second haploidentical transplant: 26 from a donor sharing the same haplotype as the first donor, and 56 from a second donor sharing a different haplotype than the first donor. One hundred four patients received their first allograft from a non-haploidentical (HID) donor (62 MSD, 24 MUD and 8 UCBD). Of these, 78 received a haploidentical allograft at the second transplant. Thus, a total of 134 patients had a new mismatched haplotype as a second allograft—78 who received a haploidentical donor allograft after a first non-haploidentical HSCT, and 56 whose second haploidentical donor shared the haplotype that was mismatched in the first transplant. The other 65 patients had a second donor that did not harbor a new mismatched haplotype—34 whose second donor was HLA-matched (24 MUD, 1 different MSD, and 3 same MSD), 26 sharing the same haplotype as the first HID (2 with the same HID), and 1 unrelated umbilical cord graft (**Figure 1**).

### Conditioning-related organ toxicity, stem cell engraftment, and chimerism

Conditioning-related organ toxicity was assessed according to NCI-CTCAE.5.0. One hundred and four (52.2%) patients had Grade 0 toxicity, 58 (29.1%) patients had Grade I toxicity, 14 patients (7.0%) were classified as having Grade II toxicity, 23 patients (11.1%) had Grade III toxicity. No patient experienced Grade IV organ toxicity.

Five patients died due to early complications prior to myeloid engraftment, five patients developed primary engraftment failure, and 189 patients survived for more than 28 days following successful myeloid engraftment. The median myeloid engraftment time was 14 days (range: 9–28). Additionally, 181 patients had successful platelet engraftment at median day 13 (range: 5–36). A bone marrow aspirate evaluation could be conducted on 189 patients within 28 days after HSCT. One patient exhibited hematological recurrence and two patients were MRD-positive at the first BM morphology examination post-HSCT. All patients exhibited full donor chimerism, and the remaining 186 patients had morphologic remission and were MRD-negative.

### Acute graft-versus-host disease (aGVHD) and chronic GVHD (cGVHD)

A total of 189 patients following the allo-HSCT2 could be evaluated for aGVHD. The cumulative incidence of Grade II–IV aGVHD and Grade III–IV aGVHD was 30.7%[95%CI,26.3-35.1] and 15.14% [95%CI,11.0-19.2], respectively. A total of 164 patients following HSCT2 were eligible for cGVHD evaluation. The 2-years cumulative incidence of cGVHD was 46.5%[95%CI,40.1-52.9], and limited cGVHD and extensive cGVHD were 21.0%[95%CI,15.9-26.2] and 30.5.0%[95%CI,25.2-36.9], respectively. Comparing GVHD outcomes between those patients that received a new mismatched haplotype and those that did not, the results were similar for the 2-years cumulative incidence of (50.0%[95%CI,44.3-57.7] vs 44.3% [95%CI,34.4-54.2], P=0.229), limited cGVHD (21.3% [95%CI,16.0-26.9]vs 11.3%[95%CI,6.8-15.8],P=0.269), and extensive cGVHD(33.2% [95%CI,27.4-40.0] vs 29.2%[95%CI,19.7-38.7],P=0.361). The 2-years cumulative incidence of cGVHD was higher in patients who received CAR-T prior to allo-HSCT2 compared to those who did not receive CAR-T

(54.3[95%CI,47.8-60.7]vs 40.8%[95%CI,29.9-51.7]; P=0.041). But the 2-years cumulative incidence of limited cGVHD was similar for patients who received CAR-T prior to allo-HSCT compared to those who did not receive CAR-T (22.7%[95%CI,17.7-27.2] vs 11.3%[95%CI,6.8-15.8],P=0.134) There was a trend towards a high extensive cGVHD for patients who had prior CAR-T compared to those who did not receive CAR-T yet no significance was observed (30.7%[95%CI,23.7-37.1]vs 10.0%[95%CI,28.9-11.0]; P=0.120).

The OS rate, LFS, and relapse rate were similar among patients who experienced Grade II–IV aGVHD and those that did not have Grade II-IV aGVHD (OS: 40.7%[95%CI,30.5-50.2] vs 46.4%[95%CI,40.7-53.1], respectively; P = 0.148), (LFS: 40.7%[95%CI,34.7-46.2] vs 44.9%[95%CI,38.3-50.1], respectively; P = 0.169), (relapse rate: 14.9%[95%CI,6.6-24.6]vs 23.1%[95%CI,16.9-30.1], respectively; P= 0.542). The OS rate and LFS was higher among patients with cGVHD compared with patients without cGVHD (60.0%[95%CI,48.5-71.5]vs 39.5%[95%CI,34.6-45.4], respectively; P = 0.051; 60.0%[95%CI, 48.5-71.5]vs 38.2%[95%CI,33.6-45.1], respectively; P = 0.045). Relapse rates were lower among patients with cGVHD compared with patients without cGVHD (0% vs 27.9%[95%CI,20.0-35.8], respectively; P = 0.052) The OS rate and LFS were also higher among patients with limited cGVHD (OS, 58.6%[95%CI,43.1-74.1] vs 31.7%[95%CI,23.8-39.6], respectively; P=0.009) and (LFS, 54.4%[95%CI,39.6-69.3] vs 30.6%[95%CI,23.4-39.2], respectively; P = 0.008) A lower CIR was also associated with limited cGVHD (26.8%[95%CI,16.4-37.2] vs 60.4[95%CI,55.6-64.9], respectively; P = 0.009), but there were no differences in the OS rate, LFS or CIR among those patients who experienced extensive cGVHD compared to those that did not (OS: 42.5%[95%CI,34.0-51.0]vs 46.0%[95%CI,42.5-50.5], respectively; P = 0.875), (LFS: 42.5%[95%CI,34.0-51.0]vs 43.6%[95%CI,39.0-48.1], respectively; P = 0.659) (CIR: 38.6%[95%CI,22.6-54.6] vs 33.5%[95%CI,28.1-38.9], respectively; P = 0.220).

### Survival and risk factor analysis

**Figure 2:**
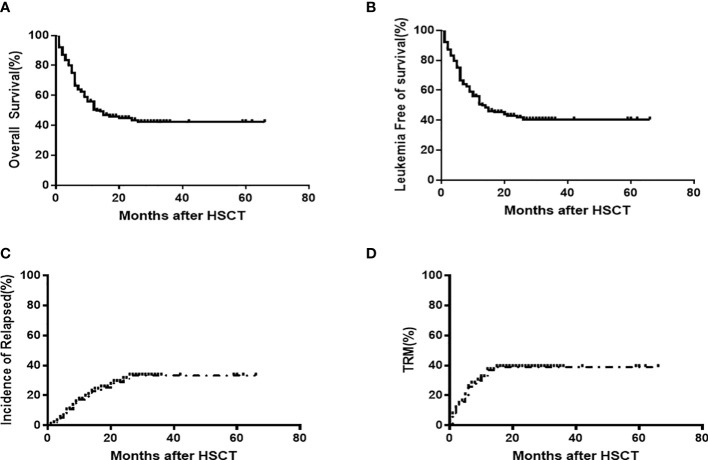
OS (43.8% [95%CI,39.7-48.0]) **(A)** and LFS 42.1% [95%CI,38.6-47.8] **(B)**, CIR(30.0%[95%CI,26.4-34.7] **(C)** and TRM 38.5%[95%CI,34.7-42.3] **(D)** outcomes following allo-HSCT2 among 199 patients.

The survivor median follow-up time was 24 months (range: 6-98), the 2-years OS and LFS were 43.8% [95%CI,39.7-48.0]and 42.1% [95%CI,38.6-47.8], respectively, and the 2-year CIR and TRM were 30.0%[95%CI,26.4-34.7] and 38.5%[95%CI,34.7-42.3], respectively (**Figures 2A–D**). Using a Cox regression analysis, patients who received CAR-T relapsed following allo-HSCT1, a CR/MRD- disease status at HSCT1 and HCT-CI (score 0) prior to allo-HSCT2, CR/MRD- disease status prior to allo-HSCT2, and those who had an HSCT2 donor with a new mismatched haplotype had better OS and LFS outcomes compared to patients without any of these characteristics (**Table 3**). Using a Cox regression multivariate analysis revealed that patients who had 1) CR/MRD- disease status, 2) HCT-CI score 0 and 3) donor with new mismatched haplotype prior to allo-HSCT2 had better OS and LFS outcomes compared to those without any of these characteristics (**Table 4**).

**Table 3:** Univariate analysis of pre-allo-HSCT2 variables associated with outcomes (Probability%[95%CI]).

	OS		LFS		CIR		TRM	
	%(95%CI)	P value	%(95%CI)	P value	% (95%CI)	P value	%(95%CI)	P value
	43.8% [39.7-48.0]		42.1%[38.6-47.8]		30.0%[26.4-34.7]		38.5%[34.7-42.3]	
Age at allo-HSCT2 (vs)		0.536		0.572		0.455		0.963
<18-y	39.5%[33.5-45.5]		39.5%[33.5-45.5]		35.3%[28.0-42.6]		38.4%[32.2-44.8])	
≥18-y	46.7%[40.9-54.5]		43.2%[37.3-49.1]		25.7%[19.3-32.1]		37.2%[32.1-42.2]	
Donor type at HSCT		0.163		0.415		0.268		0.505
MSD	47.5%[39.0-56.0]		34.4%[28.9-39.9]		17.0%[11.3-23.4]		35.3%[28.1-42.5]	
MUD	51.0%[38.0-63.2]		51.0%[38.0-63.2]		35.0%[22.8-48.8]		21.1%[13.4-29.5])	
HID	40.7%[34.6-45.8]		37.7%[32.6-42.8]		10.0%[8.2-12.3]		44.6%[39.1-50.0]	
Received CAR-T cell therapy for relapse after HSCT1		0.022		0.022		0.041		0.546
Yes	54.5%[49.1-59.9]		51.7%[45.9-57.5]		16.3%[11.6-22.0]		37.8%[32.8-43.2]	
NO	34.4%[28.9-39.9]		34.4%[28.9-39.9]		43.8%[36.6-51.0]		36.7%[31.3-42.1]	
Interval time between HSCT1 and allo-HSCT2		0.299		0.404		0.637		0.474
<12 mon.	38.5%[32.7-44.3]		38.5%[32.7-44.3]		30.6%[23.7-37.5]		43.8%[37.6-50.0]	
≥12 mon.	48.4%[42.7-54.1]		47.6%[42.0-53.2]		28.4%[22.4-34.8]		33.1%[28.9-38.0]	
Time to relapse after HSCT1		0.334		0.510		0.069		0.406
<9 mon.	40.8%[35.4-46.2]		40.8%[35.4-46.2]		35.7%[29.3-42.1]		36.1%[31.4-41.6]	
≥9 mon.	47.4%[41.2-53.6]		46.6%[40.6-52.6]		22.9%[16.0-29.8]		40.2%[35.6-45.7]	
Type of disease		0.108		0.095		0.014		0.875
Myeloid malignancy	37.0%[30.6-43.4]		37.0%[30.6-43.4]		40.7%[33.6-47.8]		35.7%[29.6-41.6]	
Lymphatic malignancy	50.7%[43.7-56.7]		46.3%[40.6-52.0]		24.3%[18.6-31.0]		38.4%[33.2-43.6]	
Disease status at HSCT1		0.031		0.064		0.008		0.629
CR/MRD neg	57.3%[49.5-66.1]		51.0%[46.5-55.6]		21.2%[17.4-26.0]		36.8%[32.2-41.4]	
CR/MRD pos and NR	20.5%[12.6-28.4]		20.5%[12.6-28.4]		54.0%[42.1-66.9]		55.0%[40.5-62.6]	
Presence of TP53 mutation pre-HSCT2		0.703		0.740		0.893		0.757
YES	41.0%[28.0-54.0]		41.0%[28.0-54.0]		32.2%[16.1-48.3]		39.1%[27.5-51.6])	
NO	44.2%[40.0-48.5]		42.4%[38.1-46.7]		13.8%[10.9-16.8]		41.4%[35.6-48.9]	
Occurrence of Grade I∼II aGVHD post- HSCT1		0.074		0.072		0.354		0.076
YES	34.3%[27.6-41.0]		32.5%[25.9-39.4]		36.7%[27.4-46.0]		45.2%[40.2-52.2]	
NO	48.3%[43.1-53.4]		48.3%[43.1-53.4]		26.9%[21.5-32.4]		37.5%[33.5-43.5]	
Occurrence of Grade III∼IV aGVHD post- HSCT1		0.650		0.640		0.256		0.053
YES	36.1%[29.6-43.1]		36.1%[29.6-43.1]		33.7%[24.6-43.1]		47.9%[42.7-55.1]	
NO	45.7%[41.3-51.5]		43.0%[38.3-48.9]		29.5%[24.5-35.8]		35.8%[31.6-40.0]	
Occurrence of limited cGVHD post-HSCT1		0.882		0.894		0.638		0.783
YES	41.4%[32.2-50.6]		41.4%[32.2-50.6]		22.1%[13.2-31.1]		42.2%[32.2-50.2]	
NO	44.3%[39.5-48.8]		42.2%[37.6-46.8]		32.2%[27.9-37.5]		37.8%[34.8-44.0]	
Occurrence of extensive cGVHD post-HSCT1		0.955		0.925		0.929		0.682
YES	43.1%[34.1-53.0]		43.1%[34.1-53.0]		24.3%[15.5-33.1]		45.9%[35.9-55.9]	
NO	41.0%[37.1-45.1]		38.9%[34.8-42.8]		30.2%[25.9-35.5]		35.8%[31.6-40.0]	
Extramedullary disease at recurrence pre- HSCT2		0.223		0.150		0.565		0.021
YES	45.4%[38.7-52.7]		45.4%[38.7-52.7]		20.5%[14.8-26.8]		42.2%[35.2-49.4]	
NO	45.0%[40.0-55.0]		42.6%[37.9-47.7]		36.0%[10.3-42.3]		45.6%[41.5-50.2]	
Donor type at allo-HSCT2		0.115		0.076		0.019		0.979
MSD	35.0%[5.6-43.7]		35.0%[5.6-43.7]		37.7%[25.0-47.0]		35.6%[29.6-42.3]	
MUD	47.3%[38.0-56.6]		42.5%[33.0-53.0]		36.8%[25.2-48.4]		21.1%[15.6-28.0]	
HID	45.1%[40.6-49.6]		45.1%[40.6-49.6]		27.0%[22.0-32.0]		44.6%[39.4-50.0]	
Conditioning regimen pre-allo-HSCT2		0.204		0.463		0.650		0.232
TBI-based	39.8%[32.1-47.5]		39.8%[32.1-47.5]		30.5%[22.1-39.1]		35.6%[29.8-42.2]	
Bu -based	45.2%[40.4-50.0]		42.6%[37.4-47.5]		30.7%[24.7-36.6]		39.0%[34.2-43.8]	
Mel-based	53.3%[30.0-77.1]		53.3%[30.0-77.1]		33.3%[21.0-59.5]		25.0%[13.4-35.7]	
New mismatched haplotype at allo-HSCT2		0.041		0.022		0.013		0.154
YES	51.0%[46.0-55.8]		50.5%[45.8-54.8]		23.4%[18.2-28.6]		26.3%[21.6-31.0]	
NO	39.0%[33.5-45.5]		32.8%[26.8-39.7]		44.6%[34.8-54.4]		40.0%[34.6-46.8]	
Donor gender matching at allo-HSCT2		0.991		0.865		0.924		0.357
M to M	41.5%[34.5-49.0]		41.5%[34.5-49.0]		35.9%[26.5-45.3]		44.4%[37.8-50.1]	
M to F	45.3%[34.3-53.1]		45.3%[34.3-53.1]		35.7%[26.4-44.9]		32.2%[26.2-37.2]	
F to F	44.3%[33.4-54.1]		44.3%[33.4-54.1]		15.2%[9.8-21.8]		48.9%[38.1-59.6]	
F to M	44.5%[36.4-52.4]		41.7%[34.6-49.6]		25.2%[18.9-32.8]		37.4%[30.4-44.8]	
Donor relationship at allo-HSCT2		0.442		0.351		0.709		0.765
Parents	46.1%[41.0-51.4]		46.1%[41.0-51.4]		35.9%[26.5-45.3]		36.0%[31.3-41.3]	
sibling	47.1%[33.9-61.7]		47.1%[33.9-61.7]		30.0%[23.4-40.7]		30.9%[19.7-42.5]	0.765
child	34.7%[23.5-45.9]		34.7%[23.5-45.9]		24.2%[16.4-32.8]		44.2%[34.2-54.2]	
Collateral series	43.8%[27.3-60.3]		43.3%[27.3-60.3]		24.8%[16.5-33.3]		35.8%[21.4-49.2]	
MUD	45.9%[36.7-55.1]		41.3%[32.0-50.6]		26.8%[8.0-36.3]		33.3%[26.7-41.6]	
Disease status pre-allo-HSCT2		0.000		0.000		0.000		0.193
CR/MRD-	56.6%[51.5-61.5]		53.3%[48.1-58.5]		14.1%[10.0-18.9]		37.6%[32.6-43.6]	
CR/MRD and NR	22.0%[16.5-28.5]		22.0%[16.5-28.5]		57.6%[47.8-67.5]		46.8%[37.5-56.1]	
Time from diagnosis to allo-HSCT2		0.250		0.352		0.112		0.993
<28 m	40.2%[34.8-45.6]		40.2%[34.8-45.6]		31.7%[25.5-37.9]		40.4%[36.1-46.2]	
≥28 m	48.8%[42.9-54.7]		47.6%[40.7-53.3]		26.1%[20.5-32.7]		35.1%[30.0-41.0]	
HCT-CI pre-allo-HSCT2		0.000		0.000		0.001		0.000
score 0	53.4%[48.9-58.2]		53.4%[48.9-58.2]		20.6%[16.5-25.0]		32.5%[27.9-37.2]	
score ≥1	25.5%[4.5-46.3]		25.0%[4.5-46.3]		48.9%[29.8-67.0]		50.0%[25.4-75.1]	

OS, overall survival; LFS, leukemia free survival; CIR, cumulative incidence of relapsed; TRM, treatment-related mortality. Neg, negative; Pos, positive.

**Table 4:** Multivariate analysis of pre-alloHSCT2 variables associated with outcomes.

Outcomes	HR (95% confidence interval)	P value
OS		
Disease status at alloHSCT2 (CR/MRD- vs CR/MRD+ plus NR)	1.484 (1.182-1.862)	0.001
HCT-CI pre-allo-HSCT2 (score 0 vs≥1)	1.709 (1.292-2.261)	0.000
With a new mismatched haplotype at allo-HSCT2 (Yes vs No)	1.440 (1.040-1.994)	0.028
LFS		
Disease status at allo-HSCT2 (CR/MRD- vs CR/MRD+ plus NR)	1.511 (1.200-1.904)	0.000
HCT-CI at allo-HSCT2 (score 0 vs≥1)	1.646 (1.241-2.184)	0.001
With a new mismatched haplotype at allo-HSCT2 (Yes vs No)	1.536 (1.022-2.308)	0.039
Relapse		
Disease status at allo-HSCT2 (CR/MRD- vs CR/MRD+ plus NR)	2.640 (1.787-3.901)	0.000
Relapsed time after HSCT1(<9m vs ≥9m)	0.460 (0.229-0.922)	0.029
With a new mismatched haplotype at allo-HSCT2 (Yes vs No)	2.977 (1.510-5.866)	0.002
TRM		
HCT-CI at allo-HSCT2 (score 0 vs≥1)	1.845 (1.330-2.560)	0.000

**Figure 3:**
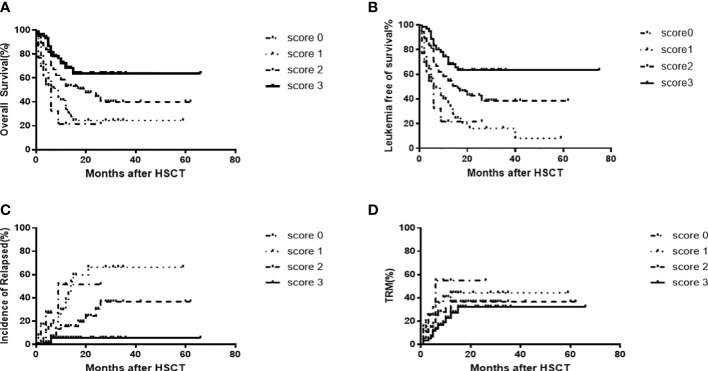
OS[score 3(63.3% [95%CI,56.6-70.0]) vs score 2(43.9%[95%CI,36.1-50.5]) vs score 1(24.2% [95%CI,18.4-30.6]6.7%)vs score 0(20.0%[95%CI,8.3-32.0]),P=0.0001)] **(A)** and LFS[score3(63.8%[95%CI,56.6-70.0]) vs score 2(43.9%[95%CI,36.6-50.5]) vs score 1(18.3%[95%CI,12.3-24.7]) vs score0 (20.0%[95%CI,8.3-32.0]), P=0.0001] **(B)** CIR [score 3 (5.5%[95%CI,2.4-8.6]) vs score 2(30.0%[95%CI,21.2-38.8]) vs score 1(65.5%[95%CI,54.6-76.4]) vs score0(51.5% [95%CI,30.6-73.3]), P=0.0001] **(C)** and TRM [score 3(32.9%[95%CI,26.2-39.6]) v score2(35.6%[95%CI,29.6-41.6]) vs score1(40.5%[95%CI,33.9-47.6]) vs score0(55.9%[95%CI,37.1-74.0]), P=0.007] **(D)** outcomes following allo-HSCT2 among 199 patients, according to prognostic score.

Next, we developed a predictive scoring system using the three favorable factors revealed by our multivariate analysis, and established a prognostic scoring system (**Table 5**). Patient outcomes, according to the presence or absence of these three favorable prognostic factors are shown in **Figure 3**.

**Table 5:** Prognostic scoring system.

Score favorable variables	Score	Number of patients
Disease status at allo-HSCT2		
CR/MRD+ and NR	0	80
CR/MRD-	1	119
HCT-CI pre-allo-HSCT2		
score ≥1	0	72
score 0	1	127
2nd donor with new mismatched haplotype	1	
NO	0	65
YES	1	134

Overall score is defined as the sum of the scores for each favorable factor. Four groups were defined as follows: score of 0 (n = 13), 1 (n = 56), 2 (n=74), and 3 (n = 60).

### Relapse, TRM and follow-up

The last follow-up for all patients was May 1, 2022. Of the 189 patients who could be evaluated by bone marrow aspirate within 28 days after HSCT, one patient showed hematology recurrence and two patients were MRD-positive at the first BM morphology examination post-HSCT2. The other 189 patients had morphologic remission and were MRD-negative. At the last follow-up, 37 patients experienced leukemia relapse (2 MRD, other had morphological recurrence). The median relapse time was 8 months (range: 1– 28) post-HSCT2. The two patients who were MRD+ received rapid tapering of immunosuppressants but finally had hematological recurrence. Of the morphological recurrences, only two patients (1 B-ALL and 1 T-ALL) who had received prior CAR-T cell therapy survived. The 2-years CIR was 30.0%[95%CI,26.4-34.7]. By univariate analysis, the pre-allo-HSCT2 variables associated with a higher CIR were 1) no prior history of CAR-T cell therapy for allo-HSCT1 relapse, 2) median time to relapse following HSCT1 of ≥8 months, 3) NR disease status at HSCT1, 4) CR/MRD+ and NR disease status at HID-HSCT2, 5) HCT-CI score≥1 prior to allo-HSCT2, 6) HSCT2 donor that was not a new mismatched haplotype (**Table 3**). Yet, our multivariate analysis showed that only patients with the following characteristics had a higher CIR: 1) CR/MRD+ disease status and NR, 2) a median time to relapse of < 8 months after HSCT1, and 3) HSCT2 donor that was not a new mismatched haplotype (**Table 4**).

Up to the last follow-up time, 102 patients died, including 67 patients that died as a result of TRM. The median time to death was 150 days (range: 11-28) post-HSCT2. The 30-day, 180-day, and 2-year cumulative incidence of TRM was 72%[95%CI,5.3-9.1],14.8%[95%CI,12.3-17.3], and 30.3%[95%CI,25.7-15.4], respectively. The patients whose conditioning-related organ toxicity ≥Grade II, 2-year cumulative incidence of TRM were higher than those with Grade 0 and Grade I groups (70.5%[95%CI,61.5-79.0] vs 51.5%[95%CI,42.5-70.2]vs 15.6%[95%CI,12.1-19.1], P=0.012).

Causes of death included severe infection (n=29), GVHD only (n=13), thrombosis microvascular disease after transplantation (n=10), viral pneumonia (n=6), hepatic sinusoidal syndrome (n=3), intracranial hemorrhage (n=1), acute renal failure (n=1), post-transplant lymphoproliferative disorder(n=1), acute myocardial infarction (n=1), acute sudden cardiac death (n=1), and drug-induced encephalopathy(n=1). Univariate and multivariate analyses of variables associated with TRM showed that a HCT-CI score ≥1 was the only risk factor for TRM. Compared to patients with a prognostic score of 2, 1, or 0, those patients with an prognostic factor score of 3 had a lower CIR (5.5%[95%CI,2.4-8.6]vs 30.0%[95%CI,21.2-38.8]vs 65.5%[95%CI,54.6-76.4] vs 51.5% [95%CI,30.6-73.3], P=0.0001) and a lower TRM (32.9%[95%CI,26.2-39.6] vs 35.6%[95%CI,29.6-41.6] vs 40.5%[95%CI,33.9-47.6]vs 55.9%[95%CI,37.1-74.0], P=0.007) (**Figures 3C, D**).

## Discussion

Prognosis of patients with acute leukemia relapsed after HSCT1 is very poor, and standard therapeutic approaches for the patients have yet to be defined. A second HSCT (HSCT2) might be the best option for the patients yet this is only possible in a select subset of patients mainly due to high rates of toxicity and relapse. Therefore, it is important to identify prognosis factor to identify those patients who could maximally benefit from an allo-HSCT2. G Andreola et al. identified three such favorable factors (1) CR at HSCT2, 2) an interval from first transplant to relapse of ≥10 months and 3) inclusion of TBI in the HSCT2 conditioning regimen) that may favorably influence LFS and OS among HSCT2 recipients. Patients with all three favorable factors had a 10-year OS rate of 36% ± 10% and an LFS of 25% ± 9%, whereas patients showing no favorable factors all died prior to the 5^th^ year (24).

Yunsuk Choi et al. divided patients based on two favorable prognostic factors (CR/CRi at HSCT2 and remission ≥6 months after HSCT1) into three groups: Group 1 (both prognostic factors) patients showed a 38.3% OS probability at 2 years in contrast to patients in Group 3 (no prognostic factors) patients who had a median OS of only 1 month (25). Still, this study lacks information on optimal second donor selection. Additional studies on optimal patient selection and identification of HSCT donor types that increase the probability of long-term survival after allo-HSCT2 are essential and need to be conducted.

In our current study, we presented a multivariate analysis of pre-transplantation factors in which we identified 1) CR/MRD- disease status, 2) HCT-CI score 0 and 3) donor with new mismatched haplotype prior to HSCT2 as three prognostic factors, that when present, may result in better OS and LFS among hematological malignancy patients receiving allo-HSCT2. The patients with all three positive prognostic factors exhibited a superior 2-year actuarial OS (63.8%[95%CI,57.2-70.4]), LFS (63.8%[95%CI, 57.2-70.4]), a lower CIR (5.6%[95%CI,2.5-8.7]) and TRM (32.3% [95%CI,25.7-38.9]), whereas patients without any of these positive prognostic factors had a lower 2-year actuarial OS (20.0%[95%CI,7.8-32.2]) and LFS (20.0%[95%CI,7.8-32.2]) and higher CIR (55.5%[95%CI,52.4-58.6]) and TRM (58.1% [95%CI,41.0-75.2]).

Prior reports have demonstrated that a CR status prior to HSCT2 is an independent prognostic factor that results in optimal outcomes (9–12). The response rate of patients with relapsed hematological malignancies who have received a transplant is low when these patients are subsequently treated with conventional approaches including chemotherapy. How to get these patients into effective remission following transplantation relapse remains a difficult problem. Currently, there are no consensus, unified treatment strategies for these patients.

In our study, we observed that the majority of patients (n = 101) received CAR T-cell therapy and achieved a CR prior to allo-HSCT2, and that the majority of them (88.1%) had lymphatic malignant hematological diseases. CAR-T cell therapy has revolutionized outcomes for patients with relapsed/refractory (R/R) B-cell hematological malignancies, resulting in high CR rates of 81% to 90% for bridge-to-allo-HSCT. Many studies have confirmed that CAR-T therapy has beneficial efficacy outcomes for other R/R hematological malignancies patients as well (21, 26–33). More recent reports, including studies from our center showed that CAR T-cell therapy can be effective for relapsed B-cell acute lymphoblastic leukemia patients following HSCT, and that a high CR rate can be achieved after CAR T therapy (34–37).

In our current study, we observed that 75 of the patients failed to respond to chemotherapy and/or a target drug and then received CAR T-cell therapy, and that 25 patients directly received CAR-T therapy following HSCT1 relapse. All the patients who received CAR-T cell therapy prior to allo-HSCT2 achieved CR (9 patients who were also MRD+) except for four patients. In univariate analyses, those patients who received CR T-cell therapy had better OS and LFS and lower CIR compared to those patients who did not receive CAR T-cell therapy. Lymphoid malignant hematological disease patients had a lower CIR rate than myeloid malignant hematological diseases although in multivariate analysis there were no differences in OS, LFS, and CIR. We believe that these outcomes may be related to the limited number of cases in this study, as we can assume that patients who have received CAR-T have a better chance of achieving CR/MRD negativity than those who received traditional chemotherapy, and this is consistent with prior studies (38, 39). As more immunotherapies have become available, we anticipate that there will be more candidates for a second HSCT with improved performance and remission status, ultimately leading to better outcomes for patients who receive a second HSCT.

Another crucial question is whether switching to a different donor for the second allograft would result in improved outcomes. In most studies to date, surprisingly, changing donor for a second allo-HSCT did not have any impact on either DFS or OS (9, 13, 14 40–42). This may be because the attempted enhancement of the GVT effect by switching donor might be affected by the toxicity that a second allo-HSCT confers. Unlike previous studies, in our study, nearly all patients (97.4%) switched to a different donor. In 134 cases (67.3%), the new donor shared a new mismatched haplotype, which means that patients use a second haploidentical donor after failure of an HLA-matched allograft, or shared a different haplotype in the case of relapse after haplo-HSCT. Univariate and multivariate analyses showed that a donor with a new mismatched haplotype resulted in better OS, LFS and a lower CIR, however, donor type did not appear to be associated with OS, LFS or CIR outcomes.

We assumed that if loss of heterozygosity is a mechanism for relapse after allo-HSCT, then utilizing a second donor allograft that recognizes the recipient haplotype shared with the first donor as non-self may improve antitumor activity and thus LFS, even in the absence of tumor haplotype loss. We also hypothesized that utilizing a donor with a different haplotype match might enhance an allogeneic antitumor effect by having a T- cell repertoire with a greater potency or efficacy for specific tumor neoantigens. In the case of a failed HLA matched transplant, switching to a different HID may be beneficial by increasing major histocompatibility mismatch. Our results are in line with those reported by Imus et al. which showed that an allograft with a new mismatched haplotype may improve outcomes after a second BMT for relapsed hematologic malignancy patients in the setting of haploidentical-HCT with post-transplant cyclophosphamide (17). Therefore, we hypothesize that for a second transplant, it is not simply that a different donor should be used, but rather an HID that should share a new mismatched haplotype if the first donor was haplo allograft. Vago,et al suggested that major HLA mismatch provides an important anti-leukemic function (43). Unfortunately, due to various limitations, we lacked complete information about patients to test for loss of heterozygosity, and we could not explore this hypothesis further in our study.

In our analysis, HCT-CI before allo-HSCT2 was the only factor related to TRM, as those with a HCT-CI score ≥1 had higher TRM compared to those with a HCT-CI score 0. These results are consistent with prior reports showing that a poor patient performance status before allo-HSCT2 increased TRM (9, 10, 13, 14). An interval from the first transplant to relapse, interval from HSCT1 to HSCT2, a change in the conditioning regimen from HSCT1 to HSCT2, and occurrence of aGVHD and cGVHD after HSCT1 are all factors previously reported to impact HSCT2 outcomes. Yet we did not detect these factors in our analyses. Many previous studies have reported that cGVHD post-HSCT is related to favorable outcomes. Consistent with these prior results, in our study, patients with cGVHD and limited cGVHD had high OS, LFS and lower CIR, but not in patients with extensive cGVHD. We observed that the incidence of extensive cGVHD was a little high, which we speculated that it may be related to rapid reduction of immunosuppressant. As most of patients received CAR-T prior to allo-HSCT2, they had a higher trend of developing extensive cGVHD although statistical significance was not observed compared to those without CAR-T. The results are consistent with our published paper by Zhao et al. from our center (21), which compared the incidence of GVHD for patients who received allo-HSCT after achieving CR from CAR-T vs chemotherapy. Zhao’s paper demonstrated that the CAR-T cohort had a higher incidence of Grade II-IV acute graft-versus-host disease (aGVHD 48.1% [95% CI: 46.1-50.1%] vs. 25.6% [95%CI: 25.2-26.0%]; p=0.016) than the chemotherapy group. The incidence of Grade III-IV aGVHD was similar in both groups (11.1% vs.11.5%, p=0.945). The overall incidence of chronic GVHD in the CAR-T group was higher compared to the chemotherapy group (73.3% [95%CI: 71.3-75.3%] vs.55.0% [95%CI: 54.2-55.8%], p=0.107), but the rate of extensive chronic GVHD was similar (11.1% vs.11.9%, p=0.964).

In conclusion, relapse after HSCT carries a high risk of poor outcomes for patients with hematological malignancies. However, there is some hope for selected patients to have long-term survival and to potentially be cured. The findings of our current study indicate that patients are likely to benefit from a second HCT after a leukemia relapse when they achieve CR/MRD-, an HCT-CI score of 0 prior to allo-HSCT2, and have a second donor with a new mismatched haplotype. As additional, novel immunotherapies including additional CAR-T become available, patients that relapse after HSCT1 are more likely to achieve remission without the added toxicities associated with aggressive conventional chemotherapy. Although the present study was neither prospective nor randomized, the risk of systemic bias among patients who have a related haploidentical donor and those who do not is unlikely, as we have not used a degree of match as a primary factor in donor choice. Still, randomized trials are needed for optimization of strategies for the treatment of hematological malignancy patients who have relapsed after a first allo-HSCT. Adapting strategies to each individual patient might be the most optimal approach.

## Data availability statement

The original contributions presented in the study are included in the article/supplementary material. Further inquiries can be directed to the corresponding authors.

## Ethics statement

The studies involving human participants were reviewed and approved by The Ethics Committee of Hebei Yanda Lu Daopei Hospital. Written informed consent to participate in this study was provided by the participants’ legal guardian/next of kin.

## Author contributions

YL designed the study. YL, J-PZ, Y-LZ, MX, R-JX, X-YC, Z-LW, J-RZ, and D-YL conducted the transplantation for patients. J-FY and XZ performed the CAR-T cell infusion for enrolled patients. YL collected the data, perform the data analysis and wrote the manuscript. D-PL and PL revised the manuscript. All authors contributed to the article and approved the submitted version.
